# Mineral elements and essential oil contents of *Scutellaria luteo-caerulea Bornm*. *&* *Snit*

**Published:** 2014

**Authors:** Mohammad Nikbin, Nasrin Kazemipour, Malek Taher Maghsoodlou, Jafar Valizadeh, Masood Sepehrimanesh, Amene Davarimanesh

**Affiliations:** 1 *Department of Chemistry, Faculty of Science, University of Sistan and Baluchistan, Zahedan, I. R. Iran*; 2 *Department of Biology, Faculty of Science, University of Sistan and Baluchistan, Zahedan, I. R. Iran*; 3 *Department of Biochemistry, School of Veterinary Medicine, Shiraz University, Shiraz, I. R. Iran*

**Keywords:** *Atomic absorption*, *Essential oils*, *Gas chromatography mass Mineral elements*, *Spectrometry*, *Spectrophotometry*

## Abstract

**Objective:** *Scutellaria luteo-caerulea Bornm. & Snit. *is one of the species of genus *Scutellaria*, within the family of the *Lamiaceae*, that is used for immune system stimulation and antibacterial effects in traditional medicine in Iran. The aims of this study were to analyze essential oils and mineral element contents of leaves of *S. luteo-caerulea* in flowering stage of development.

**Materials and Methods:** The essential oils were obtained by hydrodistillation of the leaves of *S. luteo-caerulea and *were analyzed by gas chromatography mass spectrometry (GC/MS). Moreover, microwave digestion with atomic absorption spectrophotometry were used for the mineral elements assay.

**Results:** Ninety-seven constituents were detected. Between them, the major components were trans-caryophyllene (25.4%), D-germacrene (7.9%), and linalool (7.4%). Determination of mineral elements showed that the highest minerals were Ca^2+^ (65.14±1.95 µg/ml) and K^+^ (64.67±3.10 µg/ml).

**Conclusion:** Presence of different essential oils and rich sources of Ca^2+^ and K^+^ candidate this plant as an auxiliary medication in different diseases, but more complementary researches are needed about its potency and side effects.

## Introduction

In recent years, medicinal plants have been widely used in the treatment and prevention of diseases because they have lower cost and fewer adverse effects in the body. The genus *Scutellaria* is a diverse and widespread genus within the family of the Lamiaceae (the mint family). They have over 350 species, commonly called skullcaps, and are found worldwide from Siberia to the tropics of South and North America, on the islands of Japan, and throughout a large part of Europe and Asia (Cole et al., 2007 ▶). This is a very distinctive genus in several morphological characters; perhaps the most obvious is the little crest (scutellum, literally a little shield) across the top of the calyx, the origin of the generic name (Michigan Flora Online,http://michiganflora.net/genus.aspx?id=Scutellaria). 

The extracts of this genus possess antitumor (Dai et al., 2011 ▶; Fang et al., 2012 ▶; Yin et al., 2004 ▶; Yu et al., 2007 ▶), hepatoprotective (Lin and Shieh, 1996 ▶), antioxidant (Ye and Huang, 2012 ▶; Yuan et al., 2011 ▶), anti-inflammatory (Jung et al., 2012 ▶; Zhang et al., 2012 ▶), anticonvulsant (Liu et al., 2012 ▶), antibacterial (Lu et al., 2012 ▶; Pant et al., 2012 ▶), and antiviral (Tayarani-Najaran et al., 2012; Zandi et al., 2012 ▶) effects. 

**Figure 1:**
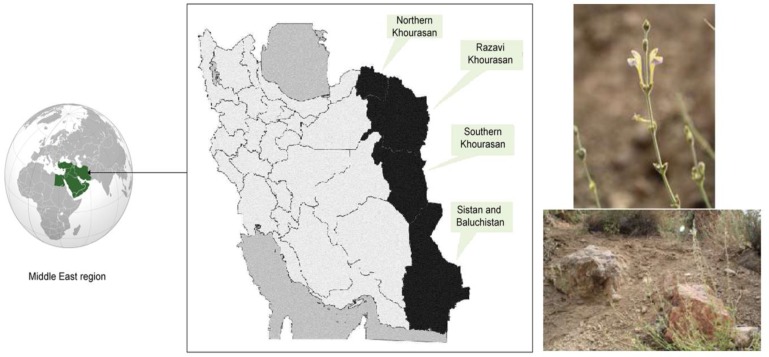
Geographical distribution of *S. luteo-caerulea* in Iran. This plant has a yellow corolla with blue-violet edges.

*S. luteo-caerulea Bornm. & Sint*. is found in the most region of Iranian plateau, such as Turkmenistan, and Iran. This species is very similar to *S. multicaulis*, but distinct according to the color and size of corolla and shape of the leaves (Bor, 1970 ▶). In Iran, it is grown in the eastern provinces including Northern, Razavi, and Southern Khorasan and Sistan and Baluchistan and locally named Boshghabi Eshghabadi (Bor, 1970 ▶). Geographical distribution and shape of this Persian plant are shown in Figure 1. 

Essential oils, also known as ethereal oils, are aromatic and largely volatile compounds. They are commonly extracted by steam distillation or solvent extraction and are usually devoid of long-term genotoxic risks (Bakkali et al., 2008 ▶; Benchaar et al., 2008 ▶). The essential oils of only a few species of *Scutellaria *such as *S. albida *ssp.* albida*, *S. sieberi*, *S. rupestris *ssp*. adenotricha*, *S. barbata*, *S. lateriflora*, *S. galericulata*,* S. parvula*,* S. baicalensis,* and *S. rubicunda subsp. Linnaeana* have been investigated (Shang et al., 2010 ▶). 

On the other hand, mineral elements play important roles in biological reactions and have structural functions. Therefore, scientists attempt to determine their concentration levels with different methods such as atomic absorption spectrometry (Hicsonmez et al., 2009 ▶). Nevertheless, no data exist concerning the essential oil composition and mineral elements contents of *luteo-caerulea Bornm. & Snit. *

Beacause of the most beneficial effects of the therapeutic plants are due to mineral contents or essential oils and lack of any data about this plant species, the purpose of the present investigation is to evaluate the essential oils and mineral elements of the leaves of *S. luteo-caerulea Bornm. & Snit.*

**Experimental procedure**

*Plant material*

*S. luteo-caerulea Bornm. & Snit.* was collected from Taftan of Sistan and Baluchistan province in Iran (GPS coordinates: 61.20816, 28.22529) during the spring season (May, 2010). All plants were in the flowering stage of developing and the taxonomic identification of each plant was confirmed by the Biology Department of University of Sistan and Baluchistan, Zahedan, Iran. The plant also matched the digital herbarium of Botanical Garden and Botanical Museum Berlin-Dahlem, Freie University, Berlin (http://ww2.bgbm.org/herbarium/(Barcode:B100241801/ImageId:278532). 

*Extraction of essential oil*

Bulked plant's leaves were used as the crude source, dried in the shade and powdered by the grinder. One hundred twenty gram of dried powder was exposed to hydrodistillation for 3 h using a Clevenger-type apparatus. The obtained essential oil was collected and anhydrous sodium sulfate was used to absorb the small amount of water containing essential oil. The essential oil was then stored at 4 °C until use.

*Essential oil analysis*

The essential oil was analyzed by GC/MS. GC analyses were performed using an Agilent 6890 GC, equipped with a HP-5 capillary column, 30 m length, 0.25 mm I.D. and 0.25 μm stationary phase film thickness, and an Agilent 5973 mass selective detector. For GC-MS detection, an ESI system with the ionization energy of 70 eV was used. Helium (99.999%) was used as the carrier gas, at the flow rate of 1 ml/min. The injection port temperature was set at 250 °C, column temperature was initially kept at 40 °C for 1 min, and then gradually increased to 240 °C at the rate of 3 °C /min. The components were identified by comparing their mass spectra with those in the GC/MS library and literature (Adams, 2001 ▶) and by comparing their relative retention times with those of authentic samples on the HP-5 MS capillary column (Table 1).

*Flame atomic absorption spectroscopy (FAAS) with microwave digestion*

In order to measure the concentration of mineral elements such as Ca^2+^, K^+^, Na^+^, Mg^2+^, Mn^2+^, Cr^3+^, and Fe^2+^, method described by Rechcigl and Payne (1990) ▶ was used. Briefly, 0.5 g of dried powdered sample was digested with 10 ml of concentrated nitric acid and then was placed inside a domestic microwave oven. The sample was irradiated at a 900 W power and 250 °C temperature for 10 min. Then, 5 ml of concentrated HCl was added and irradiation was continued for another 5 min. After digestion, the vessel was cool and then 10 ml of double distilled water was added and the mixture was filtered by Whatman No. 42 filter paper and diluted with double distilled water to a final volume of 100 ml. The solution was used for elemental analysis by atomic absorption spectrometer PU 9100X (Philips Scientific).

**Statistical analysis**

The results of the three replicates of mineral element contents were pooled and expressed as mean ± standard deviation (SD). One-way analysis of variance (ANOVA) and Tukey were carried out using SPSS version 16. Significance was accepted at p<0.05.

## Results

Hydrodistillation of the dried leaves of *S. luteo-caerulea* yielded 0.33% (v/w) of a yellowish essential oil. Ninety-seven compounds, representing 89.04% of the oil, were identified. Quantitative and qualitative analytical results are shown in Table 2. The essential oil consisted mainly of sesquiterpene hydrocarbons and oxygenated monoterpene. Trans-caryophyllene (24.8%), germacrene-D (7.9%), α-humulene (4.9%), and patchoulene(4.7%) were the main sesquiterpene hydrocarbons, whereas linalool (7.4%) and pulegone (1.1%) were the main oxygenated monoterpenes. 

**Table 1:** Authentic samples on the HP-5 MS capillary column that used for calculation of retention indices

**No.**	**Compounds**	**Formula**	**RI**	**t**_R_ (min)
**1**	n-Hexane	C_6_H_14_	600	3.166
**2**	n-Heptane	C_7_H_16_	700	4.617
**3**	n-Octane	C_8_H_18_	800	7.329
**4**	n-Nonane	C_9_H_20_	900	11.373
**5**	n-Decane	C_10_H_22_	1000	16.249
**6**	n-Undecane	C_11_H_24_	1100	21.39
**7**	n-Dodecane	C_12_H_26_	1200	26.456
**8**	n-Tridecane	C_13_H_28_	1300	29.830
**9**	n-Tetradecane	C_14_H_30_	1400	35.925
**10**	n-Pentadecane	C_15_H_32_	1500	39.321
**11**	n-Hexadecane	C_16_H_34_	1600	44.442
**12**	n-Heptadecane	C_17_H_36_	1700	47.021
**13**	n-Octadecane	C_18_H_38_	1800	51.321
**14**	n-Nonadecane	C_19_H_40_	1900	55.621
**15**	n-Eicosane	C_20_H_42_	2000	59.921
**16**	n-Heneicosane	C_21_H_44_	2100	64.221
**17**	n-Docosane	C_22_H_46_	2200	68.521
**18**	n-Tricosane	C_23_H_48_	2300	72.821
**19**	n-Tetracosane	C_24_H_50_	2400	77.121
**20**	n-Pentacosane	C_25_H_52_	2500	81.421
**21**	n-Hexacosane	C_26_H_54_	2600	85.721
**22**	n-Heptacosane	C_27_H_56_	2700	90.021

RI: retention inex, t_R_: retention time

**Table 2:** Chemical composition and percentage of essential oil of *Scutellaria luteo-caerulea*.*

**No.**	**Compounds**	**%**		**RI**	**t**_R_ ** (min)**	
**1**	Hexanal	< 0.1		702	4.670	
**2**	Octane	< 0.1		722	5.212	
**3**	Cyclohexene oxide	< 0.1		746	5.855	
**4**	2-Hexenal	0.5		755	6.112	
**5**	3-Hexen-1-ol	0.3		775	6.663	
**6**	2-Hexen-1-ol	< 0.1		791	7.072	
**7**	1-Hexanol	0.2		794	7.176	
**8**	Styrene	0.2		806	7.566	
**9**	Heptanal	< 0.1		812	7.817	
**10**	Tricyclene	< 0.1		845	9.149	
**11**	p-Methylbenzyl alcohol	< 0.1		850	9.368	
**12**	α-Pinene	0.1		857	9.636	
**13**	Benzaldehyde	0.4		860	9.774	
**14**	Camphene	0.1		869	10.127	
**15**	Sabinene	< 0.1		895	11.169	
**16**	β-Pinene	0.1		898	11.279	
**17**	3-Octanone	< 0.1		901	11.409	
**18**	1-Octen-3-ol	1.8		910	11.830	
**19**	β-Myrcene	< 0.1		915	12.108	
**20**	3-Octanol	0.8		923	12.456	
**21**	δ-3-Carene	< 0.1		932	12.883	
**22**	α-Terpinene	0.2		937	13.138	
**23**	p-Cimene	0.2		941	13.317	
**24**	1,8-Cineole	0.6		948	13.650	
**25**	Limonene	0.8		950	13.771	
**26**	cis-Ocimene	< 0.1		958	14.160	
**27**	Acetophenone	0.5		965	14.485	
**28**	Trans-β-Ocimene	0.4		969	14.666	
**29**	γ-Terpinene	0.3		977	15.045	
**30**	α-Terpinolene	0.1		1002	16.372	
**31**	Nonanal	0.1		1010	16.782	
**32**	Linalool	7.4		1026	17.567	
**33**	Benzene (1,3-dimethyl-2-butenyl)-	0.3		1039	18.250	
**34**	Camphor	0.8		1042	18.421	
**35**	Phenoprene	0.8		1054	19.034	
**36**	6-[(Z)-1-Butenyl]-1,4-cycloheptadiene	0.1		1059		19.280
**37**	2-Methylnorbornene	< 0.1		1070		19.857
**38**	4-Terpineol	0.4		1081		20.407
**39**	α-Terpineol	0.7		1094		21.063
**40**	Cyclooctene, 4-methylene-6-(1-propenylidene)	1.1		1112		21.979
**41**	Pulegone	1.1		1128		22.807
**42**	Geraniol	0.6		1156		24.229
**43**	Borneol, acetate	0.1		1174		25.155
**44**	bicyclogermacrene	0.6		1243		27.912
**45**	Cadina-1,4-diene	1.2		1261		28.528
**46**	(-)-Cycloisosativene	0.6		1285		29.308
**47**	α-Copaene	3.1		1297		29.732
**48**	α-Longipinene	5.2		1302		29.948
**49**	trans-Caryophyllene	25.4		1332		31.806
**50**	α-Gurjunene	0.4		1343		32.459
**51**	Aromadendrene	< 0.1		1345		32.593
**52**	Valencene	0.3		1346		32.659
**53**	α-Humulene	5.1		1356		33.228
**54**	α -Cubebene	0.4		1357		33.327
**55**	β-Cubebene	0.2		1359		33.448
**56**	Epizonarene	< 0.1		1365		33.798
**57**	D-Germacrene	7.9		1374		34.323
**58**	α-Ylangene	0.4		1376		34.472
**59**	Patchoulene	4.8		1381		34.792
**60**	δ-Cadinene	0.3		1385		35.004
**61**	γ-Cadinene	0.9		1389		35.270
**62**	Pentadecane	0.5		1392		35.452
**63**	β-Himachalene	2.6		1397		35.757
**64**	Cadina-1,4-Diene	0.4		1401		35.951
**65**	α-cadinene		0.3	1406		36.140
**66**	(-)Dehydroaromadendrane		0.5	1414		36.396
**67**	3-Hexen-1-ol, benzoate	0.9		1432		37.027
**68**	β-Bisabolene	0.2		1439		37.238
**69**	Caryophyllene oxide	3.8		1449		37.590
**70**	β -Humulene	0.3		1455	37.781	
**71**	Ledene	0.5		1462	38.034	
**72**	endo-2-Methylbicyclo [3.3.1]nonane	0.6		1474	38.441	
**73**	Naphthalene, 1,2,3,4,6,8a-hexahydro-1-isopropyl-4,7-dimethyl	0.3		1500	39.308	
**74**	Aromadendrene	0.6		1504	39.529	
**75**	tau-Cadinol	1.5		1511	39.869	
**76**	t-Muurolol	1.2		1520	40.328	
**77**	α -Muurolene	0.3		1531	40.920	
**78**	Heptadecane	0.6		1567	42.750	
**79**	Mintsulfide	0.1		1569	42.829	
**80**	Octadecane	< 0.1		1666	46.135	
**81**	Neophytadiene	< 0.1		1725	48.110	
**82**	Nonadecane	< 0.1		1757	49.463	
**83**	Hexadecanoic acid, methyl ester	< 0.1		1764	49.788	
**84**	Dibutyl phthalate	< 0.1		1769	49.988	
**85**	n-Hexadecanoic acid	0.5		1809	51.703	
**86**	Eicosane	< 0.1		1831	52.640	
**87**	Linolenic acid, methyl ester	< 0.1		1883	54.884	
**88**	Phytol	0.3		1903	55.757	
**89**	Linoleic acid	< 0.1		1925	56.703	
**90**	(E)-9-Octadecenoic acid	0.7		1930	56.923	
**91**	Octadecanoic acid	< 0.1		1944	57.508	
**92**	n- Heneicosane	< 0.1		1969	58.589	
**93**	α-Farnesene	0.1		1998	59.831	
**94**	Docosane	< 0.1		2034	61.387	
**95**	Tricosane	< 0.1		2096	64.069	
**96**	Tetracosane	< 0.1		2157	66.660	
**97**	Squalene	< 0.1		2338	74.457	

* (The compounds are listed in order of their elution on HP-5).

**Table 3:** Mineral elements of leaves of *Scutellaria luteo-caerulea Bornm. & Sint*

**Elements**	**Concentration (µg/ml)**
**Ca** ^2+^	65.14 ± 1.95^a^
**K** ^+^	64.67 ± 3.10^a^
**Mg** ^2+^	7.07 ± 1.02^b^
**Mn** ^2+^	4.38 ± 1.68^b^
**Fe** ^2+^	2.79 ± 0.25^c^
**Cr** ^3+^	2.21 ± 0.23^c^
**Na** ^+^	0.27 ± 0.02^d^

Different superscript letters indicate significant differences (p<0.05).

**Figure 2:**
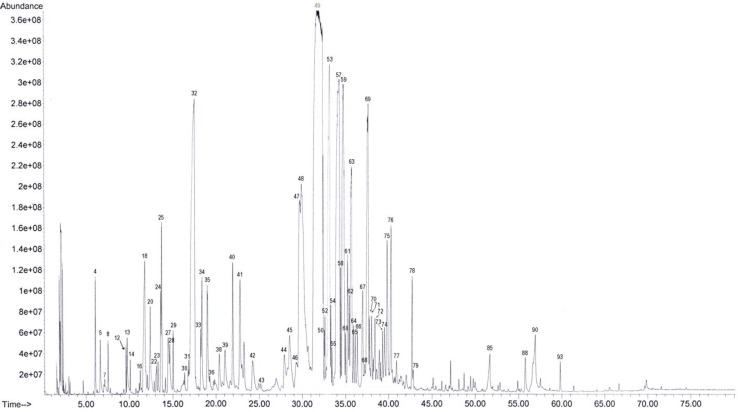
The GC-MS chromatogram of essential oils of *S. luteo-caerulea*. Only components that had higher than 0.1 % value were numbered. Component number according to Table 2.

The values of several oxygenated sesquiterpene, such as caryophyllene oxide (3.8%), tau-cadinol (1.4%), and t-muurolol (1.2%) were also significant. The GC-MS chromatogram of essential oils of *S. luteo-caerulea* was shown in Figure 2. In this figure, only components that had higher than 0.1 % value were numbered. Mineral elements of leaves of *S. luteo-caerulea *were shown in Table 3. According to our founding, Ca^2+^ (65.14±1.95 µg/ml) and K^+^ (64.67±3.10 µg/ml) had the highest concentrations followed by Mg^2+^ (7.07±1.02).

## Discussion

In this study, the therapeutic valency of the *S. luteo-caerulea* *Bornm. & Snit. *was measured by analysis of the essential oils and mineral element contents of leave part of this plant. Essential oils of *S. luteo-caerulea* *Bornm. & Snit. *had not been evaluated previously, but several studies were conducted for other species and subspecies. Skaltsa and their colleagues reported that linalool was the main compound of the oil of *S. albida *ssp*. albida* (Skaltsa et al., 2000a ▶) and found it in high amounts in other species including *S. sieberi* and *S. rupestris* ssp. *adenotricha* (Skaltsa et al., 2005b ▶). The essential oil contents of *Scutellaria pinnatifida* was evaluated by Ghannadi and Mehregan (2003). Their results demonstrated that germacrene-D and beta-caryophyllene were the most essential oils that found in the aerial parts of this widespread Iranian Skullcaps. 

In another study by Yu et al. (2004) ▶, the essential oils of leaves of *S. barbata *from China was evaluated. They reported that the main components of the oil was hexahydrofarnesyl acetone followed by 3,7,11,15-tetramethyl-2-hexadecen-1-ol, menthol and 1-octen-3-ol. Yaghmai (1988) ▶ described that β-cadinene and calamenene were the major components of the oils of *S. lateriflora *along with β-elemene, α-cubebene, and α-humulene. Caryophyllenes were the main compounds of *S. rubicund*a subsp. *linnaean*a reported by Rosselli et al. (2007) ▶. 

In another part of this study, sample preparation with microwave digestion was used for mineralization of this plant. This method was faster and easier than the old digestion method such as wet and dry ashing. Totally, seven different minerals were existed in this plant as different levels. Analytical results of all the analyzed elements in this plant were given in Table 3. The results showed high concentrations of Ca^2+^ and K^+^ and low concentration of Na^+^, Cr^3+^, and Fe^2+^ in the plant. Calcium is an essential element that is found in high concentrations in plants (Hicsonmez et al., 2009 ▶) and plays different roles such as structural, in the cell wall and membranes, a counter‐cation for inorganic and organic anions, in the vacuole, and an intracellular messenger in the cytosol (Marschner, 1995 ▶). Another main element in *S. luteo-caerulea* *Bornm. & Snit. *is K^+^. It is needed for activation of some enzymes, protein synthesis in ribosomes, turgor provision, and water homeostasis and also plays roles in photosynthesis (Maathuis, 2009 ▶). 

The content of nine mineral elements in the root, stem, and leaf of *S. baica*lensis was evaluated by Zhu et al. (2011) ▶. Their results showed that the main mineral elements in all three parts were K^+^, Ca^2+^, Mg^2+^, P^4-^, Al^3+^, and Fe^2+^. In another study, the contents of six elements, Ca^2+^, Cu^2+^, Fe^2+^, Mn^2+^, Zn^2+^, and K^+^, in five parts of planted *S. baicalensis* were determined by FAAS. They reported that Ca^2+^ in the flowers, seeds, and roots and Fe^2+ ^in the stems and leaves were the main elements (Sheng et al., 2009 ▶). Our findings are in concordance with the results of these two studies that demonstrated that Ca^2+ ^and K^+^ are found in high concentration in this genus. In summary, our study demonstrated that *S. luteo-caerulea Bornm. & Sint* was the rich sources of Ca^2+ ^and K^+ ^and had many different essential oils specially trans-caryophyllene, D- germacrene, and linalool. Further studies are required for analysis of its *in vivo* effects and to develop new drugs and therapeutic agents from essential oils of this plant.
